# Neighborhood and Environmental Context of Health and Aging: Mechanisms and Heterogeneity

**DOI:** 10.1093/geroni/igaf122.890

**Published:** 2025-12-31

**Authors:** Jeffrey Stokes, Heather Farmer, Martina Brandt

## Abstract

There is a rich literature establishing the importance of neighborhood and environmental context for successful aging, including physical, mental, and cognitive health in later life. Yet, to date, the mechanisms underlying these associations are not fully understood, nor has research thoroughly examined heterogeneity in the role(s) neighborhood and environmental context play. This research has also been limited, overlooking diversity within the U.S. as well as outside it. This symposium addresses this gap by examining implications of multiple levels of environment across diverse and international samples of older adults. Dr. Wang will assess associations of neighborhood social and built environment with ADRD diagnosis. Dr. Álvarez will discuss mechanisms linking neighborhood stressors with cognition among the aging population in Brazil. Dr. Choi will highlight aging-related immune phenotypes as potential biological mechanisms for effects of neighborhood characteristics on long-term health. Dr. Thierry focuses on the unique experience of Black adults in the U.S., examining neighborhood stress and cognitive functioning variations within and outside of the South and across urban/rural settings. Finally, Dr. Cantu examines whether environment – exposure to extreme heat – is associated with cognitive health and related behaviors among older Mexican Americans. As Discussant, Dr. Brandt will synthesize the contributions made by these papers concerning mechanisms underlying place effects on health and heterogeneity across diverse groups within the U.S. and internationally. This symposium holds the potential to shape policy efforts aimed at structural influences on successful aging and to provide scholars with a more nuanced understanding of contextual factors and health.

